# A Rare Case of Iliopsoas Muscle and Retroperitoneal Lesion Secondary to Malakoplakia

**DOI:** 10.7759/cureus.114657

**Published:** 2026-08-17

**Authors:** Juan J Zorrilla Ariza, Valentina Ulloa Zuluaga, Jose M Zorrilla Ariza, Daniela E Angulo Eraso, Jose Bravo Bonilla, Sergio D Leon Castro

**Affiliations:** 1 School of Medicine, Universidad Instituto Colombiano de Estudios Superiores de Incolda (ICESI), Cali, COL; 2 School of Medicine, Pontificia Universidad Javeriana, Cali, COL; 3 School of Medicine, Universidad del Valle, Cali, COL; 4 Pathology, Universidad del Valle, Cali, COL; 5 Surgery, Universidad del Valle, Cali, COL

**Keywords:** benign granulomatous disease, histopathology (hp), laparoscopic surgery, malakoplakia, michaelis-gutmann bodies, psoas muscles, retroperitoneal space

## Abstract

Malakoplakia is a rare, benign granulomatous inflammatory disorder caused by a defect in the phagolysosomal function of macrophages, which often mimics neoplastic or severe infectious processes. We present a case of a 43-year-old immunocompetent woman with abdominal and right inguinal pain, in whom imaging studies identified a solid retroperitoneal pelvic mass and focal involvement of the right iliopsoas muscle, highly suggestive of metastatic malignancy. Initial and definitive histopathological examination following laparoscopic resection confirmed the diagnosis by demonstrating infiltration by foamy macrophages (von Hansemann cells) with pathognomonic basophilic intracytoplasmic inclusions (Michaelis-Gutmann bodies) and positive CD68 staining. The patient had a favorable clinical course following surgical intervention and prolonged antibiotic therapy with agents exhibiting high intracellular penetration. Retroperitoneal and iliopsoas involvement in immunocompetent patients represents an exceedingly rare presentation. Timely histopathological diagnosis is essential to guide multidisciplinary management and rule out malignancy.

## Introduction

Malakoplakia is a rare entity; therefore, its epidemiological characterization is limited and is based primarily on case series and retrospective reviews. The prevalence of malakoplakia is unknown; however, more than 700 cases had been reported in the literature by 2011 [1,2].

It presents variations according to anatomical location, with genitourinary involvement, particularly bladder involvement, being more common in women, whereas cutaneous forms predominate in men. The disease primarily affects middle-aged and older adults, with a higher incidence beginning in the fifth decade of life; however, isolated pediatric cases have been described. It is more frequently associated with immunosuppression and comorbidities such as diabetes mellitus, organ transplantation, lymphoma, chronic steroid use, and alcoholism. Its name is derived from the Greek words malakos (soft) and plakos (plate) [1-4].

Macroscopically, it manifests as yellowish or whitish, flat, soft lesions that often exhibit central umbilication and may mimic neoplastic or infectious processes, making clinical diagnosis challenging. It originates from a defect in the ability of macrophages to digest bacteria, resulting in the accumulation of calcified bodies known as Michaelis-Gutmann bodies. Its etiology and pathogenesis are poorly understood; furthermore, its clinical presentation and imaging findings are nonspecific, and therefore, its diagnosis requires histopathological examination. Michaelis-Gutmann bodies can be identified with hematoxylin and eosin (H&E), periodic acid-Schiff, Prussian blue, and especially von Kossa stains, the latter providing greater sensitivity for highlighting them [3,5].

Muscular involvement usually manifests as pseudotumoral masses that require histopathological confirmation by biopsy and may mimic neoplastic processes [3,6]. Treatment is based on prolonged antibiotic therapy with agents that have adequate intracellular penetration and activity against microorganisms implicated in its pathogenesis, particularly fluoroquinolones, trimethoprim-sulfamethoxazole, and rifampicin. Antibiotic therapy should be adjusted according to microbiological results and susceptibility profiles. In immunosuppressed patients, modification of immunosuppressive therapy may be necessary. Surgical management depends on the affected organ, extent of disease, and presence of symptoms or pseudotumoral masses [3-5].

We present a case of malakoplakia involving the right iliopsoas muscle and a retroperitoneal mass in an immunocompetent patient whose clinical presentation was characterized predominantly by right-sided abdominal pain. The literature review indicates that, although cases of malakoplakia have been described, retroperitoneal involvement and, specifically, involvement of the iliopsoas muscle are exceptional findings, highlighting the rarity and relevance of this report [1,3,5-12].

## Case presentation

A female patient presented to the outpatient gynecology clinic on day one for follow-up of abnormal uterine bleeding, bringing an external transvaginal ultrasound report that identified a right adnexal mass categorized as Ovarian-Adnexal Reporting and Data System (O-RADS) 5, associated with uterine leiomyomas. She also provided a report of positive tumor markers for CA-125 and CA-19-9 (Table 1). The patient reported right lower abdominal pain radiating to the ipsilateral lumbar region, accompanied by dysuria and urinary tenesmus, and was therefore referred to the emergency department.

**Table 1:** Serum tumor marker levels at initial evaluation.

Tests	Patient values	Reference range
Alpha-fetoprotein (AFP)	0.90 ng/mL	0.5-7 ng/mL
Carcinoembryonic antigen (CEA)	0.80 ng/mL	0.0-5.2 ng/mL
Cancer antigen 125 (CA-125)	69.70 U/mL	0.0-34.99 U/mL
Cancer antigen 19-9 (CA-19-9)	139 U/mL	0.0-39 U/mL

Upon admission to the emergency department, the abdomen was soft and depressible, with superficial and deep tenderness in the right hemiabdomen, without signs of peritoneal irritation. Initial vital signs were as follows: heart rate: 108 beats/min, blood pressure: 99/60 mmHg, respiratory rate: 20 breaths/min, oxygen saturation: 97%, and axillary temperature: 36.5°C. The initial laboratory results are detailed in Table 2. The general surgery service was consulted and requested a computed tomography (CT) scan of the abdomen and pelvis.

**Table 2:** Initial laboratory findings on admission.

Tests	Patient values	Reference range
Leukocytes	10.40×10³/µL	4.23-9.07×10³/µL
Neutrophils	7.20×10³/µL	1.56-6.13×10³/µL
Lymphocytes	2.30×10³/µL	1.18-3.74×10³/µL
Hemoglobin	12.2 g/dL	11.2-15.7 g/dL
Platelets	412×10³/µL	182-369×10³/µL
C-reactive protein	7 mg/L	0-5 mg/L

**Figure 1:**
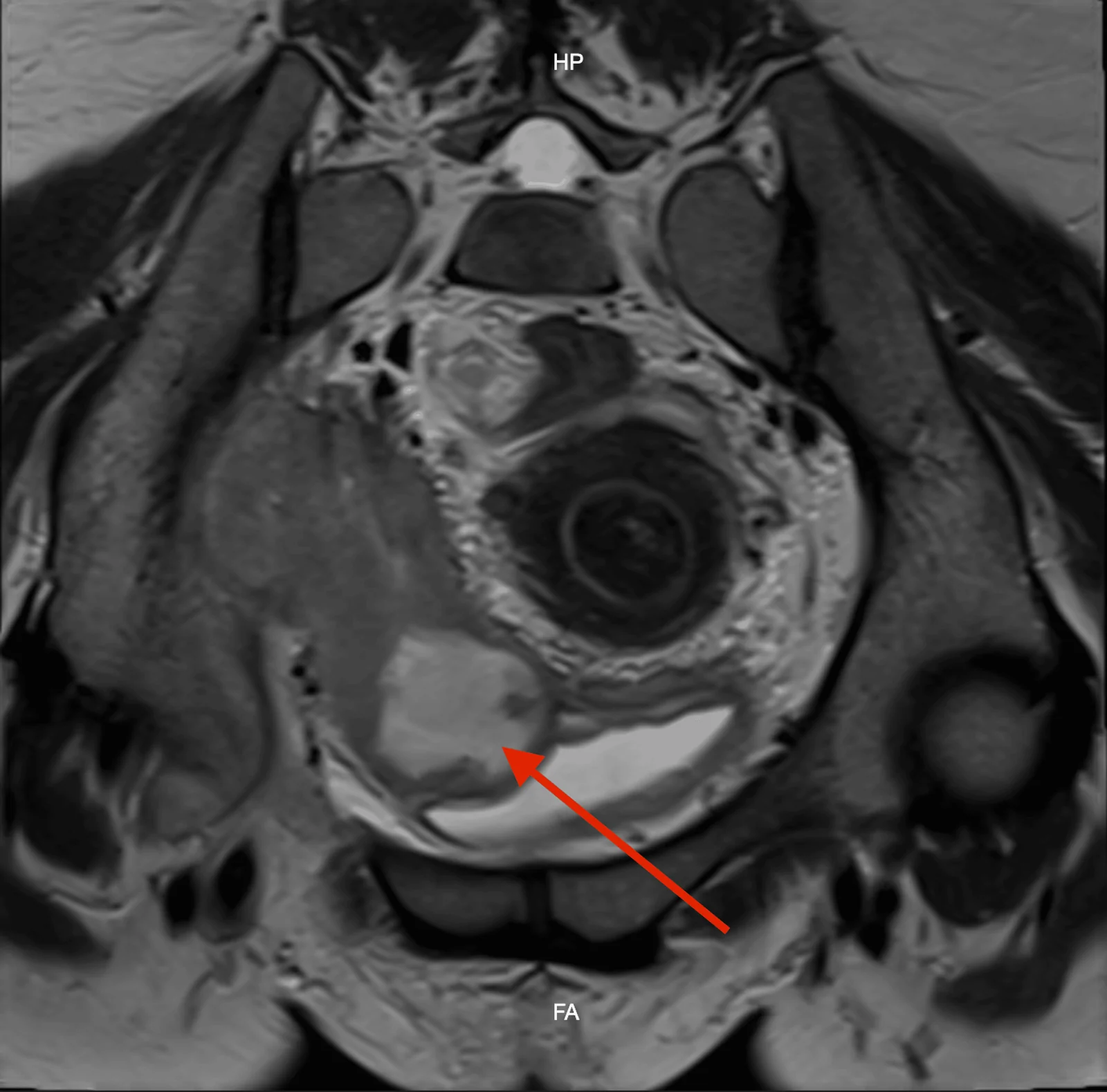
Magnetic resonance imaging of the abdomen and pelvis in the coronal plane, T2-weighted sequence. Right-sided retroperitoneal pelvic solid mass with a neoplastic appearance, indicated by the arrow. HP: head-posterior; FA: foot-anterior

**Figure 2:**
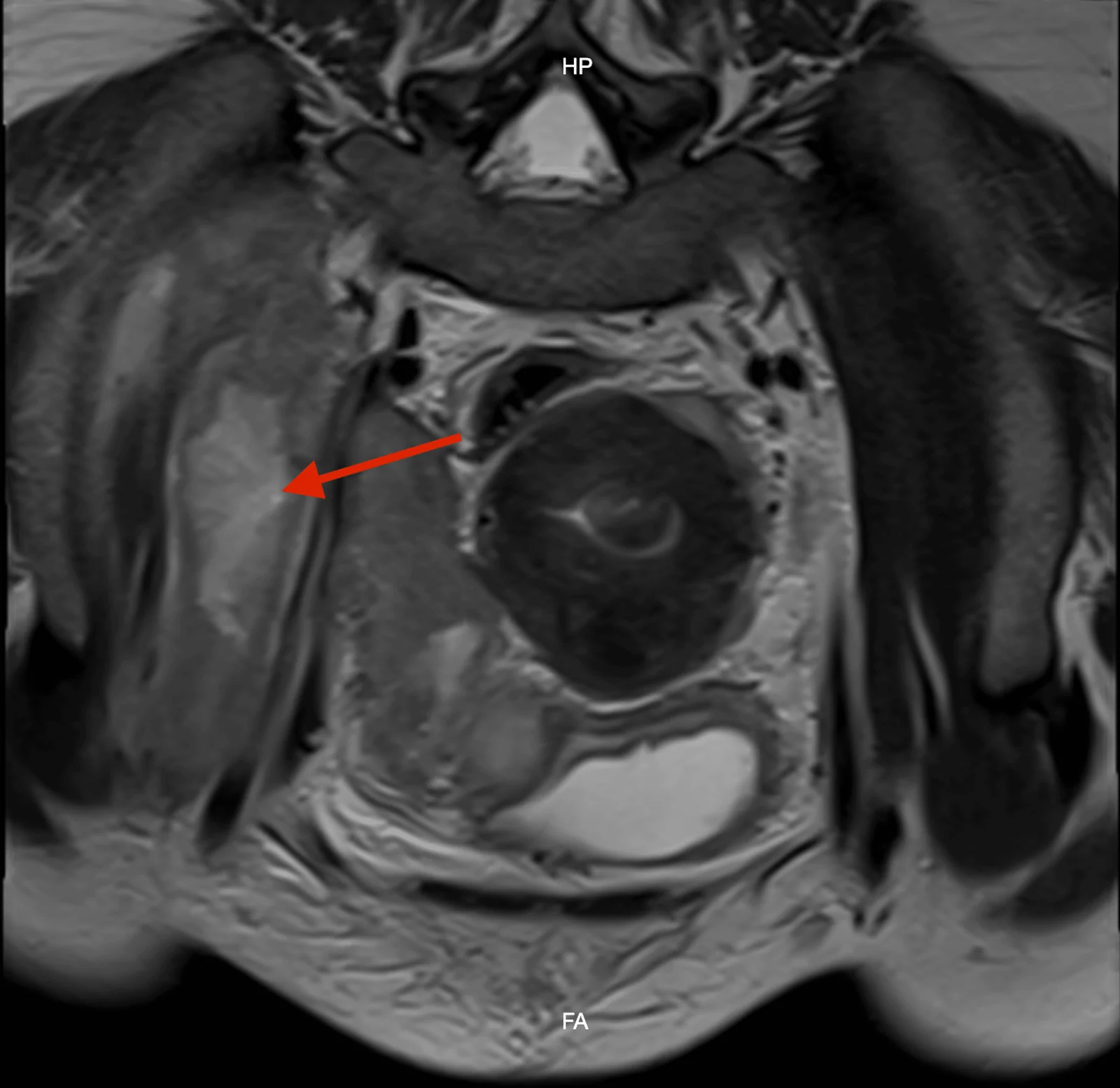
Magnetic resonance imaging of the abdomen and pelvis in the coronal plane, T2-weighted sequence. Focal solid lesion in the right iliopsoas muscle, indicated by the arrow. HP: head-posterior; FA: foot-anterior

On day three, an abdominal and pelvic CT scan was performed, demonstrating a right adnexal neoplastic process with intra- and extra-peritoneal involvement and extension to the uterine region, as well as multiple inguinal and bilateral iliac chain lymphadenopathies suggestive of diffuse infiltration. To further characterize these findings, a contrast-enhanced magnetic resonance imaging (MRI) study of the abdomen and pelvis was performed on day five, showing a right-sided retroperitoneal solid pelvic mass with a neoplastic appearance and probable metastatic lymphatic involvement of the right external iliac chain, encasing the right iliac vessels and extending anteriorly toward the space of Retzius, without a discernible cleavage plane from the bladder wall. The lesion measured approximately 12.5×5×7.3 cm (Figure 1). Additionally, a focal solid lesion was identified in the right iliopsoas muscle, consistent with metastatic involvement (Figure 2).

For this reason, on day eight, a biopsy of the right iliopsoas muscle was performed. Additionally, the patient was pain-free and hemodynamically stable (heart rate: 98 beats/min, blood pressure: 100/65 mmHg, respiratory rate: 16 breaths/min, oxygen saturation: 98%, and axillary temperature: 35.4°C). Therefore, on day nine, she was discharged from the hospital with pain management and an order for outpatient evaluation by surgical oncology.

On day 15, the histopathological examination reported an extensive polymorphonuclear infiltrate with abundant histiocytes with abundant eosinophilic and granular cytoplasm, associated with basophilic intracytoplasmic inclusions compatible with Michaelis-Gutmann bodies, thus establishing the diagnosis of malakoplakia. This diagnosis was reviewed for the first time on day 31, when she attended the surgical oncology outpatient clinic, where, based on the histopathological finding of malakoplakia, evaluation by infectious diseases and internal medicine was ordered.

On day 56, the infectious diseases service evaluated her and initiated antibiotic therapy with trimethoprim-sulfamethoxazole for 12 weeks; a purified protein derivative (PPD) test was also requested. On day 57, the internal medicine service evaluated her and considered it necessary to rule out a concomitant primary neoplasm, requesting a high-resolution chest computed tomography scan.

**Figure 3:**
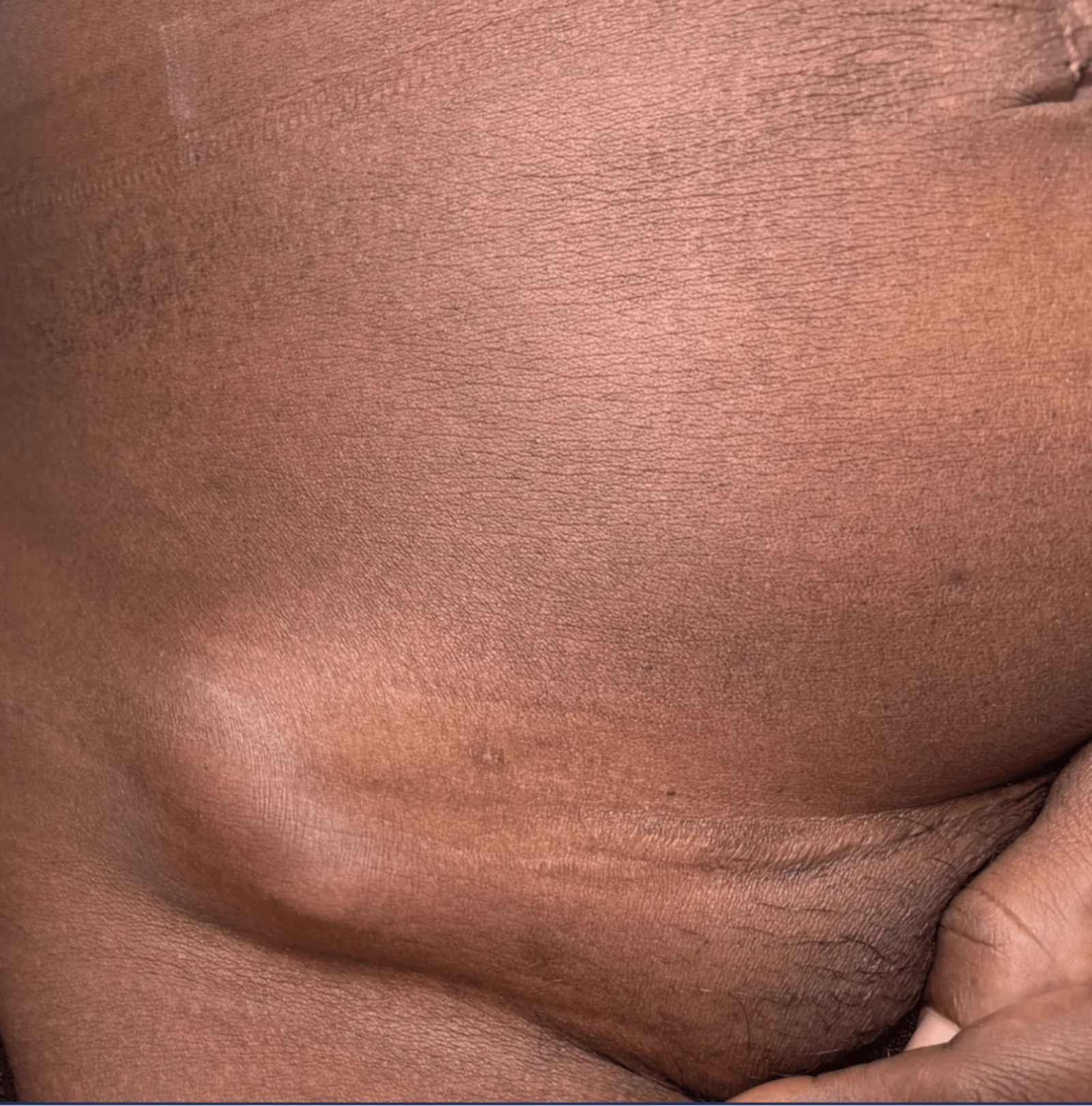
Right inguinal mass measuring approximately 5x5 cm is observed.

On day 73, the patient was readmitted to the emergency department due to exacerbation of pain, undocumented fever, and an increase in the size of the right inguinal mass, associated with local inflammatory changes. Physical examination revealed a painful mass measuring approximately 5×5 cm in the right inguinal region, adherent to the deep tissue planes, with erythema, local warmth, and rubor (Figure 3). Her vital signs were as follows: heart rate 80 beats/min, blood pressure 130/70 mmHg, respiratory rate 18 breaths/min, oxygen saturation 97%, and axillary temperature 37.1°C. Laboratory tests revealed leukocytosis and elevated C-reactive protein levels, raising a high suspicion of a superinfected lymph node lesion (Table 3). The general surgery service was consulted and requested contrast-enhanced computed tomography of the pelvis. On the same day, day 73, a soft-tissue ultrasound was performed, which showed an image suggestive of a collection in the right inguinal region with probable extension into the deep tissue planes.

**Table 3:** Laboratory findings at readmission to the emergency department.

Tests	Patient values	Reference range
Leukocytes	16.59×10³/µL	4.23-9.07×10³/µL
Neutrophils	13.50×10³/µL	1.56-6.13×10³/µL
Lymphocytes	2.54×10³/µL	1.18-3.74×10³/µL
Hemoglobin	11.8 g/dL	11.2-15.7 g/dL
Platelets	471×10³/µL	182-369×10³/µL
C-reactive protein	191 mg/L	0-5 mg/L

**Figure 4:**
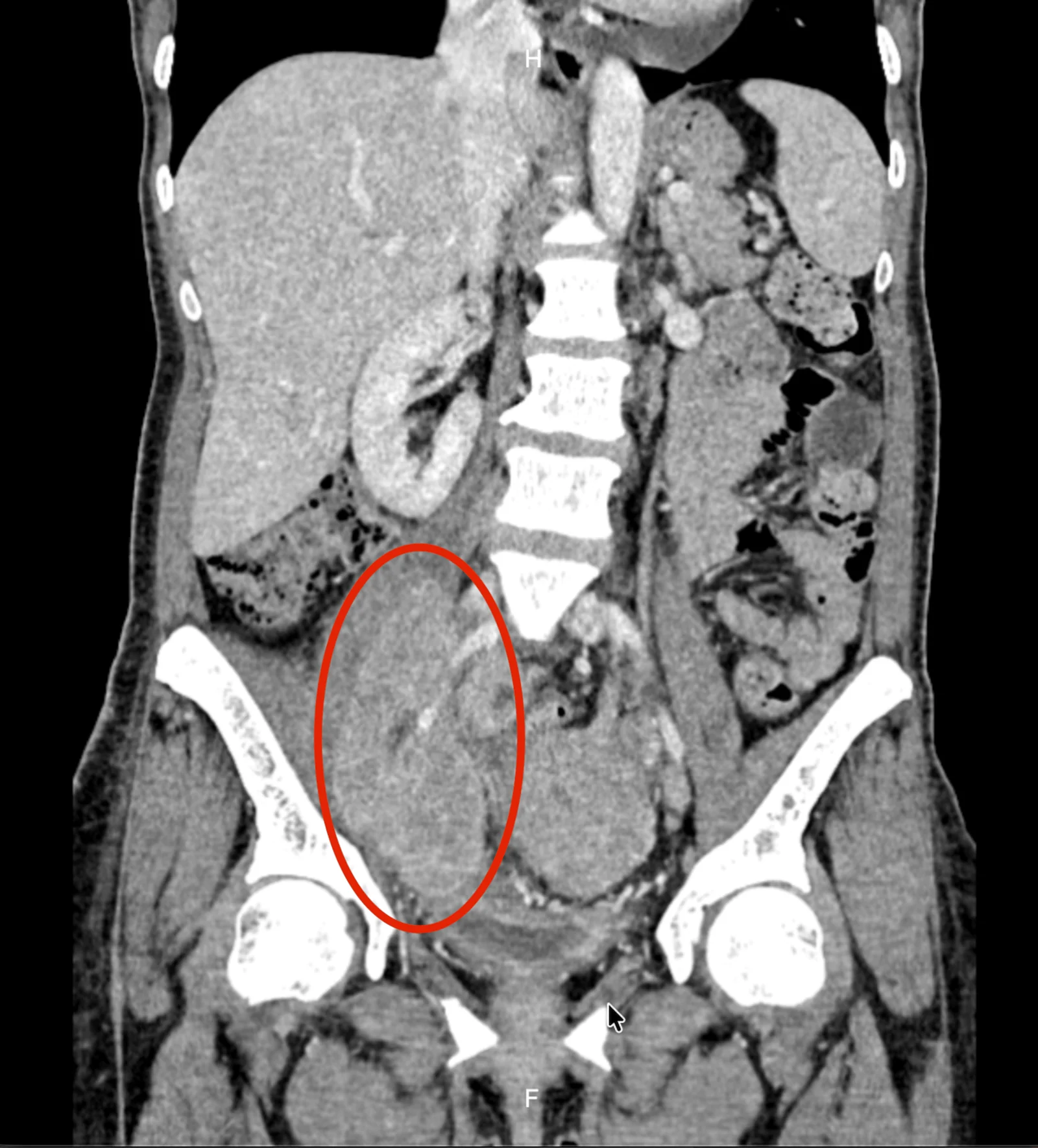
Abdominal and pelvic computed tomography in the coronal plane with intravenous contrast. Lesion suggestive of a collection extending from the right adnexal region toward the iliopsoas muscle, with heterogeneous post-contrast enhancement, indicated by an arrow. H: head; F: foot

Subsequently, a contrast-enhanced abdominal CT performed on day 76 demonstrated a lesion suggestive of a collection extending from the right adnexal region toward the iliopsoas muscle, with heterogeneous post-contrast enhancement, initially suggesting an infectious process originating from the right adnexa. Bilateral inguinal lymphadenopathy and diffuse retroperitoneal inflammatory changes in the right iliac fossa were also observed (Figure 4).

On day 76, the infectious diseases service evaluated the patient, discontinued trimethoprim-sulfamethoxazole, and initiated ceftriaxone 2 g IV every 24 h. Testing for chronic infections, including human immunodeficiency virus (HIV), syphilis serology, human T-cell lymphotropic virus type I/II (HTLV-I/II), and hepatitis B and C, was requested, and all results were negative (Table 4).

**Table 4:** Screening results for chronic infectious diseases. COI: Cut-Off Index; HTLV-I/II: human T-cell lymphotropic virus type I/II; RPR: rapid plasma reagin

Tests	Patient values	Reference range/result
Human immunodeficiency virus 1 and 2 antibodies	COI 0.24	COI <1.0, nonreactive
Hepatitis B surface antigen	COI 0.4	COI <0.89, nonreactive
Hepatitis C antibody	COI 0.0	COI <0.9, nonreactive
HTLV-I/II total antibodies	COI 0.1	COI <0.99, nonreactive
Manual RPR nontreponemal test	-	Nonreactive

Gynecologic oncology re-evaluated the patient and considered that the adnexal lesion previously categorized as O-RADS 5 corresponded to the same finding identified as malakoplakia, recommending follow-up only if malignancy was documented. On the same day, day 76, she was re-evaluated by general surgery after review of the CT findings; the service determined that there was no indication for surgical management at that time and that obtaining a new biopsy of the lesion should be postponed because of the active infectious focus, considering that she was already receiving antibiotic coverage under the care of infectious diseases. Given that the collection was small, management was provided by the enterostomal therapy service, which on day 77 obtained a culture of the lesion located in the right groin. The lesion was soft in consistency, measured 2×2 cm, and involved the superficial dermis without deep involvement (with limited ability to assess the pocket because of poor patient cooperation). Drainage of 30 cc of foul-smelling purulent material with abundant membranes was obtained.

As part of the extension workup, a high-resolution chest CT was performed on day 79, identifying a previously known 7.6-mm subsolid nodule in the anterior segment of the right upper lobe, which was already under follow-up by pulmonology, with no other pulmonary abnormalities. On day 80, an upper gastrointestinal endoscopy was performed and showed no lesions suggestive of malignancy. The surgical oncology service noted that malakoplakia is an uncommon entity and recommended further evaluation to rule out associated etiologies; a colonoscopy was performed and revealed no pathological findings.

On day 85, the patient underwent percutaneous drainage by interventional radiology and an attempted biopsy of the right retroperitoneal mass; however, the procedure could not be performed because the lesion was surrounded by large-caliber vascular structures, making the intervention unfeasible. She was re-evaluated by infectious diseases on day 87, and continuation of treatment with doxycycline and ceftriaxone was recommended, given that the main etiologic agents of malakoplakia are Enterobacteriaceae.

On day 90, microbiological studies, including polymerase chain reaction (PCR) for *Mycobacterium tuberculosis* and direct examination with potassium hydroxide (KOH), were negative. *Staphylococcus epidermidis* was isolated and was susceptible to the tested antibiotics; however, this finding was discordant with the clinical presentation, and therefore, no changes were made to the antibiotic regimen (Table 5).

**Table 5:** Microbiological and mycological study results. KOH: potassium hydroxide

Tests	Laboratory findings
KOH direct examination for fungi	Negative
*Mycobacterium tuberculosis* PCR	Not detected
Culture of fluid collection	*Staphylococcus epidermidis* - susceptible

On day 91, the patient underwent laparoscopic resection of the retroperitoneal tumor by the gastrointestinal surgical oncology service. Intraoperative findings included a pelvic adhesive process with fibrinous membranes that prevented adequate visualization of the bladder and rectouterine pouch. A lymph node conglomerate measuring approximately 5×5 cm was identified over the right iliopsoas muscle. Dissection of the lymph node conglomerate and retroperitoneal lesion was performed, and the specimens were sent for definitive histopathological examination.

On day 94, the patient showed clinical improvement, with no abdominal pain and no elevation of acute-phase reactants (Table 6). Treatment with doxycycline and ceftriaxone was continued. Finally, on day 96, the patient was evaluated by surgical oncology and internal medicine. Physical examination revealed a soft, depressible abdomen with covered surgical wounds, without evidence of discharge or bleeding, and tenderness on palpation around the lesion. In the iliac fossa, there was a lesion without evidence of active bleeding, covered with a dressing. Her vital signs were as follows: heart rate 80 beats/min, blood pressure 104/70 mmHg, respiratory rate 18 breaths/min, oxygen saturation 98%, and axillary temperature 36°C. Given her clinical stability at that time, she was discharged from the hospital with outpatient follow-up. The infectious diseases service adjusted the outpatient antibiotic regimen to sultamicillin 750 mg orally every 12 h for three months and doxycycline 100 mg orally every 12 h for three months.

**Table 6:** Laboratory findings during clinical improvement.

Tests	Patient values	Reference range
Leukocytes	5.81×10³/µL	4.23-9.07×10³/µL
Neutrophil	3.12×10³/µL	1.56-6.13×10³/µL
Lymphocyte	2.19×10³/µL	1.18-3.74×10³/µL
Hemoglobin	12.6 g/dL	11.2-15.7 g/dL
Platelet	340×10³/µL	182-369×10³/µL
C-reactive protein	3.10 mg/L	0-5 mg/L

**Figure 5:**
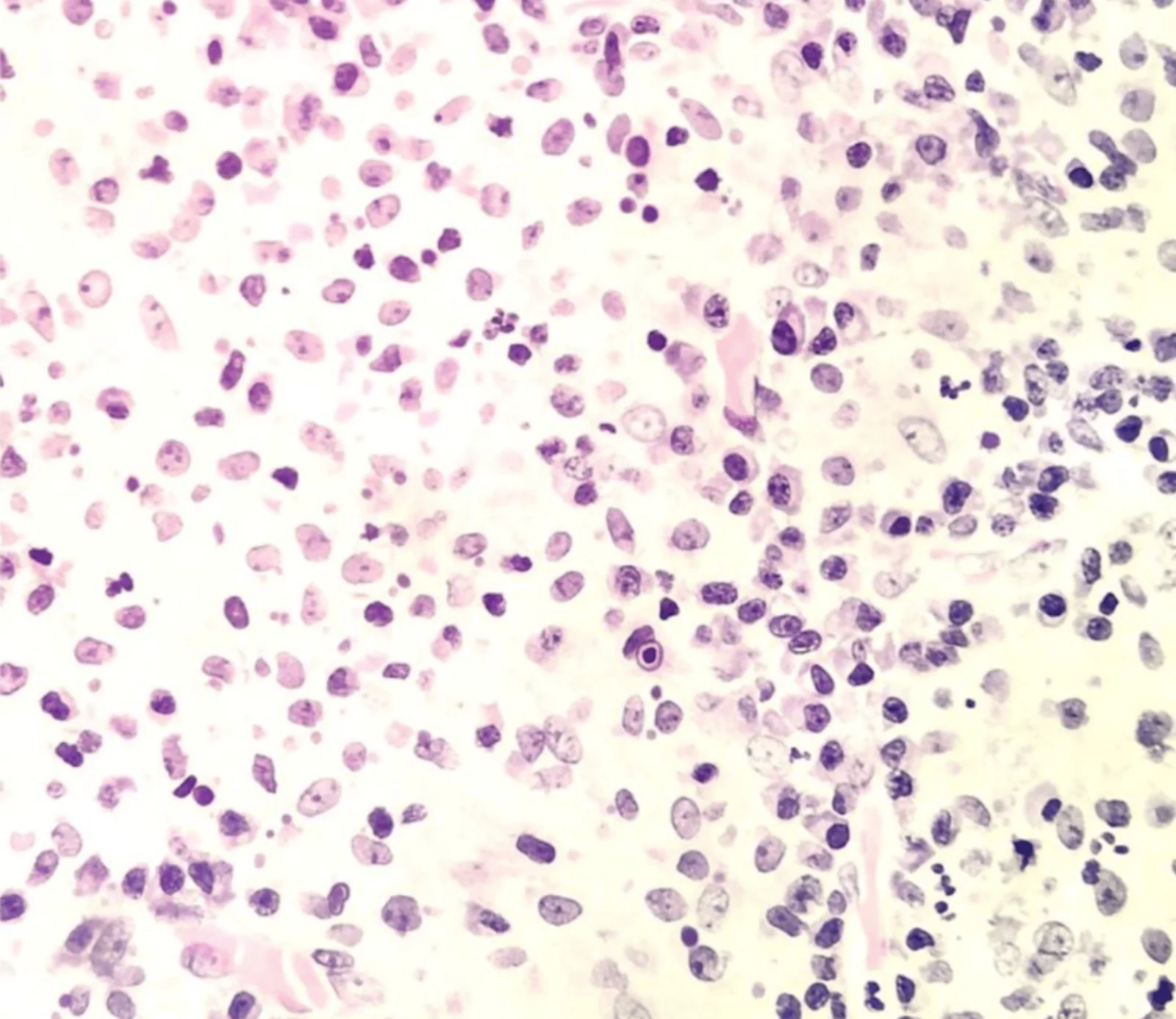
Hematoxylin and eosin stain (original magnification ×10). A rounded, intracytoplasmic body is observed within a foamy histiocyte (von Hansemann cell).

**Figure 6:**
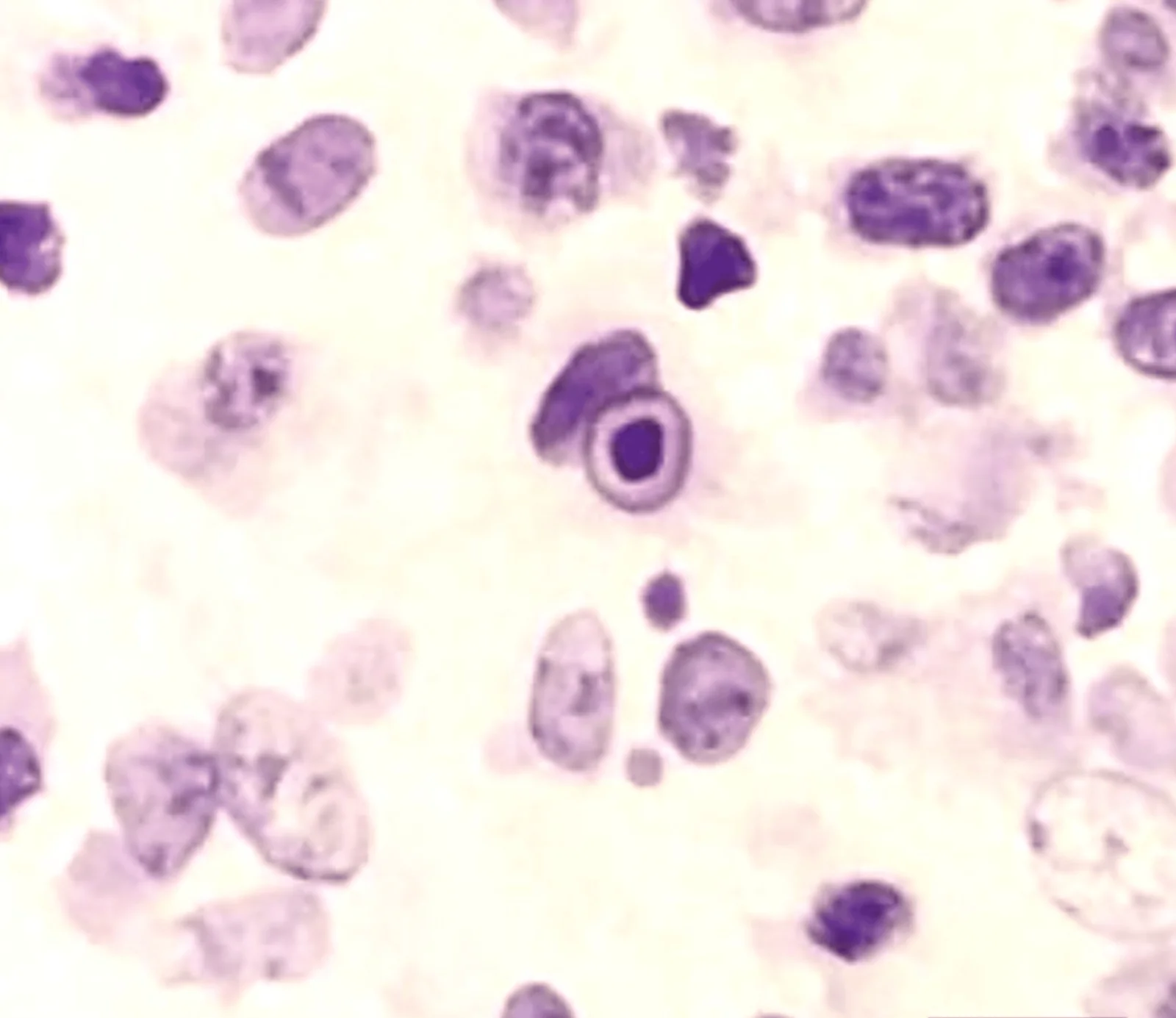
Hematoxylin and eosin stain (original magnification ×40). The structure exhibits a concentric laminated appearance with a dense basophilic center surrounded by a clear halo, producing the characteristic “target” or “owl’s eye” appearance. It typically measures 5 to 15 µm, and the surrounding cytoplasm contains abundant macrophages with granular cytoplasm.

The definitive histopathological analysis confirmed an extensive inflammatory infiltrate composed of neutrophils, plasma cells, lymphocytes, and histiocytes. Basophilic intracytoplasmic inclusions compatible with Michaelis-Gutmann bodies were identified, findings diagnostic of malakoplakia (Figures 5, 6).

**Figure 7:**
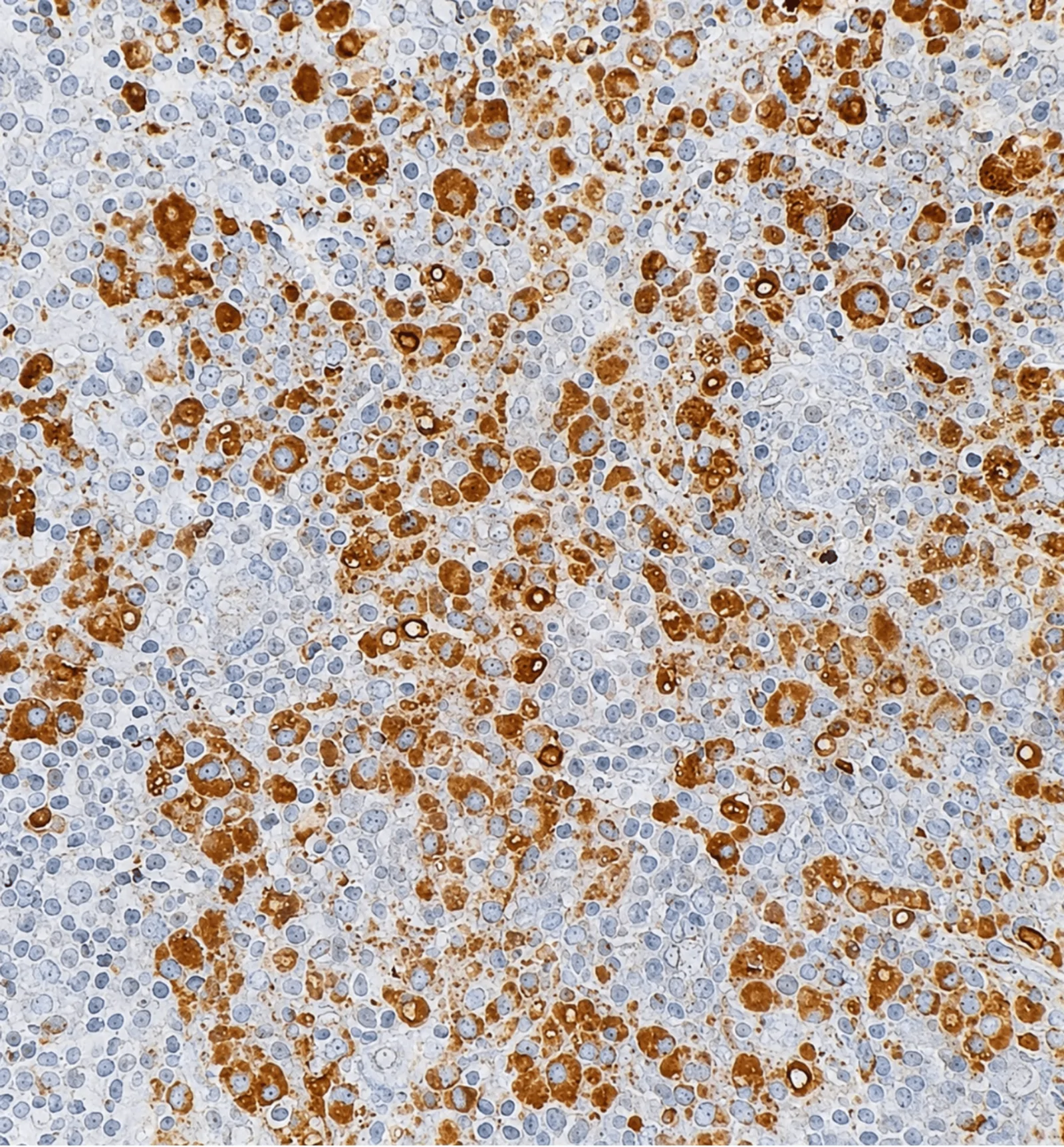
CD68 immunohistochemistry (original magnification ×10). CD68 immunohistochemical staining demonstrates a broadly and diffusely distributed population of histiocytes with strong cytoplasmic positivity. Intracytoplasmic inclusions are observed in some of the positive cells.

The immunohistochemistry report showed histiocytes (CD68+) broadly and diffusely distributed, with cytoplasmic inclusions, mature plasma cells (CD38+, CD56-), and T- and B-cell lymphoid populations (CD3+ and CD20+). The findings were negative for CK AE1/AE3, with a Ki-67 index of 40% in the germinal center. The findings were consistent with malakoplakia (Figure 7).

On day 111, gynecologic oncology evaluated the patient as an outpatient and determined that, given the benign nature of the condition, no further follow-up by that specialty was required. On day 112, infectious diseases evaluated her, with continued clinical improvement, no abdominal pain, and no elevation of acute-phase reactants. Continued treatment with doxycycline and sultamicillin was recommended. She subsequently attended follow-up with a complete blood count showing no leukocytosis, renal function within normal limits, and no abdominal pain or other symptoms (Table 7).

**Table 7:** Laboratory findings during outpatient follow-up.

Tests	Patient values	Reference range
Leukocytes	5.32×10³/µL	4.23-9.07×10³/µL
Neutrophil	2.94×10³/µL	1.56-6.13×10³/µL
Lymphocyte	2.10×10³/µL	1.18-3.74×10³/µL
Hemoglobin	12.4 g/dL	11.2-15.7 g/dL
Platelet	345×10³/µL	182-369×10³/µL
C-reactive protein	1.70 mg/L	0-5 mg/L

**Figure 8:**
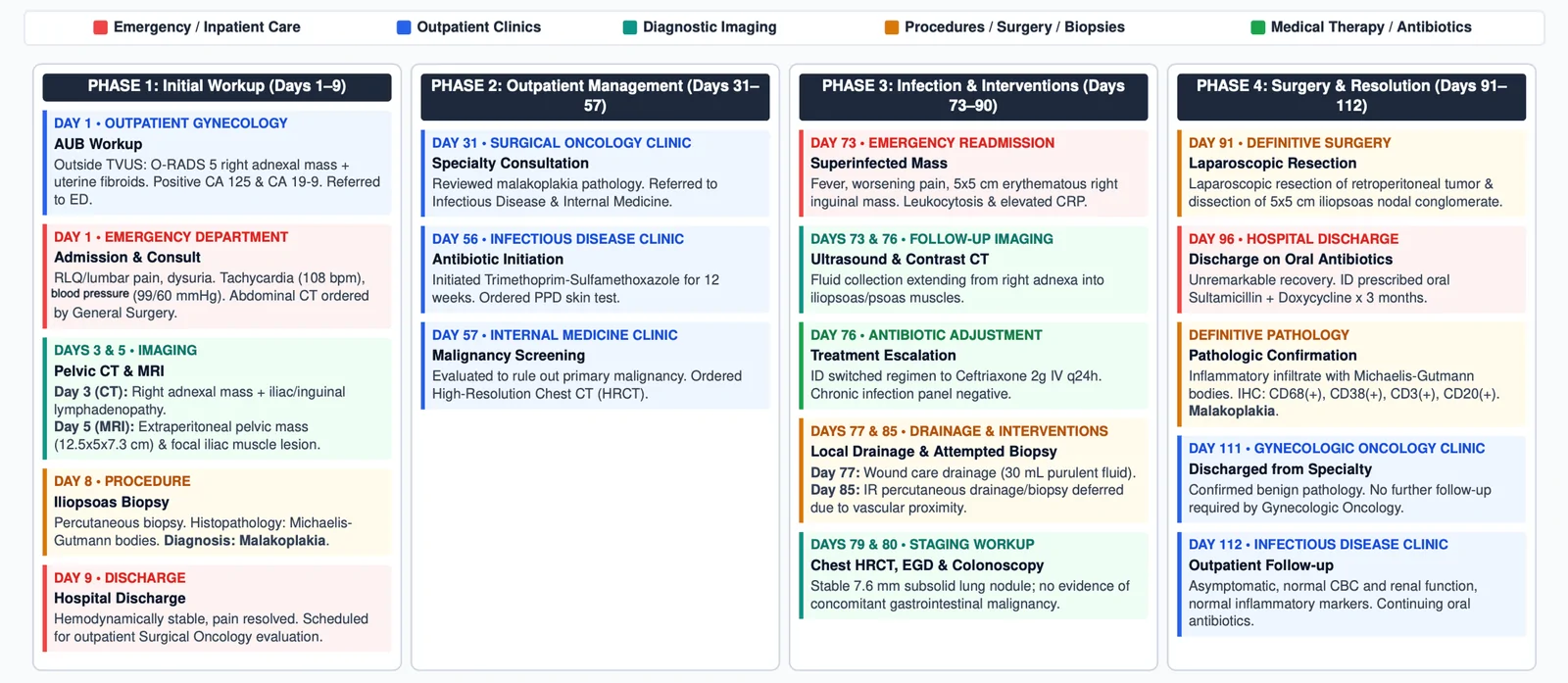
Chronological timeline of clinical management, diagnostic workup, and interventions. AUB: abnormal uterine bleeding; TVUS: transvaginal ultrasound; O-RADS: Ovarian-Adnexal Reporting and Data System; HRCT: high-resolution computed tomography; EGD: esophagogastroduodenoscopy; CRP: C-reactive protein; IHC: immunohistochemistry; ID: infectious disease; IR: interventional radiology; PPD: purified protein derivative; RLQ: right lower quadrant

Because of the complexity and prolonged clinical course, the diagnostic events, imaging studies, surgical interventions, and implemented treatment regimens are summarized chronologically in a timeline to facilitate understanding of the case (Figure 8).

## Discussion

Malakoplakia is a rare granulomatous disease first described in the early 20th century. Because of its nonspecific clinical presentation and ability to mimic infectious or neoplastic processes, it is known as a “great imitator,” posing a significant diagnostic challenge because it can be confused with more prevalent conditions [3,13,14].

The imaging findings of malakoplakia are nonspecific; up to 75% of cases may present with multifocal lesions that coalesce into masses with an infiltrative appearance, mimicking neoplastic processes or abscesses. Despite its pseudotumoral behavior, it is a benign inflammatory entity whose definitive diagnosis depends on histopathological examination because of its low clinical and imaging specificity. For this reason, it represents a diagnostic challenge in clinical practice, as it must be differentiated from multiple entities with similar presentations, including infectious processes such as tuberculosis, cryptococcosis, actinomycosis, botryomycosis, and leishmaniasis; inflammatory diseases such as Crohn's disease, sarcoidosis, and hidradenitis suppurativa; as well as tumoral or pseudotumoral lesions such as lymphoma, granular cell tumors, and xanthomas. In the present case, magnetic resonance imaging demonstrated a retroperitoneal pelvic mass with a neoplastic appearance and a focal lesion in the right iliopsoas muscle suggestive of metastatic involvement; however, histopathological examination by biopsy confirmed the diagnosis of malakoplakia [1,4,5,9,13,15,16].

Additionally, malakoplakia has classically been associated with patients with chronic diseases or immunosuppressive states, such as HIV infection, organ transplantation, immunosuppressive therapy, diabetes mellitus, tuberculosis, malignancies, and other conditions that impair the immune response [4,10]. However, in the present case, the patient was immunocompetent, highlighting the unpredictable nature of this entity and emphasizing that malakoplakia can develop even in the absence of clearly established risk factors [4,9]. This finding reinforces the need for a high index of suspicion and consideration of this entity in the differential diagnosis of pseudotumoral lesions in unusual locations.

The pathogenesis of malakoplakia is rooted in dysfunction of mononuclear phagocytes, characterized by macrophages' inability to effectively degrade internalized bacteria. This impairment in phagolysosomal activity is attributed to reduced levels of cyclic guanosine monophosphate (cGMP) and deficient release of beta-glucuronidase, resulting in altered microtubular function and lysosomal digestion. Consequently, partially digested microorganisms accumulate and trigger a chronic inflammatory response. Although malakoplakia is associated with immunosuppression and predominantly with Gram-negative bacilli, with *Escherichia coli* being the most frequently isolated organism in the genitourinary tract, followed by Klebsiella, Proteus, Pseudomonas, and biofilm-forming microorganisms such as Stenotrophomonas, the occurrence of cases in immunocompetent patients and in the absence of microbiological isolation suggests that additional etiopathogenic factors remain to be elucidated [3,13].

In the literature review, we identified a study that compiled 59 patients diagnosed with malakoplakia and described their main clinical characteristics and anatomical distribution. The most frequent site of involvement was the kidney, identified in 29 cases (49.2%), followed by the gastrointestinal tract in 11 cases (18.6%), the ureter or bladder, and the perineal/inguinal region, each in seven cases (11.9%). Less frequent locations included the abdominal wall, lung, face, liver, spleen, axilla, and prostate. However, among the reported locations, no cases with primary involvement of the iliopsoas muscle were identified, highlighting the exceptional presentation described in our report [6].

The definitive diagnosis of malakoplakia is established by histopathological examination, characterized by an inflammatory infiltrate composed of lymphocytes, plasma cells, and large foamy histiocytes (von Hansemann cells). These cells contain rounded basophilic cytoplasmic inclusions known as Michaelis-Gutmann bodies, considered pathognomonic findings and corresponding to intracellular deposits of calcium and iron secondary to phagolysosomal dysfunction. Michaelis-Gutmann bodies can be identified with hematoxylin and eosin, periodic acid-Schiff, Prussian blue, and especially von Kossa stains, which offer greater sensitivity for highlighting them. Immunohistochemistry with CD68 and CD163 markers is used to confirm the histiocytic (von Hansemann) population [3-5,13]. In our case, Michaelis-Gutmann bodies were identified on hematoxylin and eosin staining, and immunohistochemistry demonstrated CD68-positive histiocytes.

Management may involve pharmacological and surgical treatments, depending on the individual assessment of each case. Pharmacological management has evolved over time and currently focuses on antibiotics with intracellular penetration and agents that improve macrophage function. Because malakoplakia involves defective intracellular bacterial digestion, antibiotics that can enter the intracellular compartment are preferred; the most commonly used include trimethoprim-sulfamethoxazole, rifampin, and sulfonamides. The use of tetracyclines such as doxycycline and vancomycin has also been reported [7,11]. Although these studies were not specifically designed to address malakoplakia involving the iliopsoas muscle or a retroperitoneal mass, this case demonstrates that extrapolation of these antibiotic regimens can result in an adequate response, as observed in the patient’s biochemical course.

Surgical intervention in malakoplakia is reserved for selected cases, primarily those with obstructive involvement, associated collections, refractory disease, or lesions with pseudotumoral characteristics in which malignancy cannot be ruled out. In some locations, surgical resection may constitute definitive treatment, particularly when conservative management fails or there is suspicion of an associated tumor, as occurred in the present case, in which laparoscopic resection of the retroperitoneal lesion was necessary [4,17,18].

The relevance of this case lies in its unusual presentation involving the right iliopsoas muscle and a retroperitoneal mass in an immunocompetent patient. Diagnosis required a multimodal approach, including advanced imaging studies, serial biopsies, and multidisciplinary evaluation, which allowed the diagnosis to be clarified and the most appropriate therapeutic management to be established.

## Conclusions

Malakoplakia represents a significant diagnostic challenge due to its ability to mimic malignant neoplasms and complex infectious processes. Involvement of the iliopsoas muscle and retroperitoneal space in an immunocompetent patient constitutes an exceedingly rare presentation. In the presence of nonspecific imaging findings with an infiltrative and metastatic appearance, histopathological confirmation through the identification of von Hansemann cells and Michaelis-Gutmann bodies is essential for definitive diagnosis. Given the limited literature and the atypical nature of these presentations, further studies and systematic registries are needed to clarify the current epidemiology and disease behavior across different anatomical locations.

Management of this entity requires a multidisciplinary approach integrating histopathological evaluation, prolonged antibiotic regimens with high intracellular penetration, and laparoscopic surgical resection aimed at ruling out a primary neoplasm or concomitant malignancy. Timely histopathological characterization of this benign granulomatous process allows appropriate therapeutic management to be established.
